# Recent Advances in the Analysis of Functional and Structural Polymer Composites for Wind Turbines

**DOI:** 10.3390/polym17172339

**Published:** 2025-08-28

**Authors:** Francisco Lagos, Brahim Menacer, Alexis Salas, Sunny Narayan, Carlos Medina, Rodrigo Valle, César Garrido, Gonzalo Pincheira, Angelo Oñate, Renato Hunter-Alarcón, Víctor Tuninetti

**Affiliations:** 1 Department of Mechanical Engineering, Universidad de La Frontera, Temuco 4811230, Chile; f.lagos06@ufromail.cl; 2 Master Program in Engineering Sciences, Faculty of Engineering, Universidad de La Frontera, Temuco 4811230, Chile; 3 Laboratoire des Systèmes Complexes (LSC), Ecole Supérieure en Génie Electrique et Energétique ESGEE Oran, Chemin Vicinal N9, Oran 31000, Algeria; menacer_brahim@esgee-oran.dz; 4 Departamento de Ingeniería Mecánica (DIM), Facultad de Ingeniería (FI), Universidad de Concepción, Concepción 4070409, Chile; alesalas@udec.cl (A.S.); cmedinam@udec.cl (C.M.); 5 Department of Mechanics and Advanced Materials, Campus Monterrey, School of Engineering and Sciences, Tecnológico de Monterrey, Av. Eugenio Garza Sada 2501 Sur, Tecnológico, Monterrey 64849, Mexico; s.narayan@tec.mx; 6 Facultad de Arquitectura, Construcción y Medio Ambiente, Universidad Autonoma de Chile, Talca 3460000, Chile; rodrigo.valle@uautonoma.cl; 7 Department of Mechanical Engineering, Universidad del Bío-Bío, Concepción 4081112, Chile; cgarrido@ubiobio.cl; 8 Department of Industrial Technologies, Faculty of Engineering, University of Talca, Camino a Los Niches Km 1, Curicó 3344158, Chile; gpincheira@utalca.cl; 9 Department of Materials Engineering (DIMAT), Faculty of Engineering, Universidad de Concepción, Concepción 4070138, Chile; aonates@udec.cl; 10 Composite Materials Laboratory, Department of Mechanical Engineering, Universidad de La Frontera, Temuco 4780000, Chile

**Keywords:** polymer composites, fluid–structure interaction, structural fatigue, structural health monitoring, leading-edge erosion, composite structural optimization

## Abstract

Achieving the full potential of wind energy in the global renewable transition depends critically on enhancing the performance and reliability of polymer composite components. This review synthesizes recent advances from 2022 to 2025, including the development of next-generation hybrid composites and the application of high-fidelity computational methods—finite element analysis (FEA), computational fluid dynamics (CFD), and fluid–structure interaction (FSI)—to optimize structural integrity and aerodynamic performance. It also explores the transformative role of artificial intelligence (AI) in structural health monitoring (SHM) and the integration of Internet of Things (IoT) systems, which are becoming essential for predictive maintenance and lifecycle management. Special focus is given to harsh offshore environments, where polymer composites must withstand extreme wind and wave conditions. This review further addresses the growing importance of circular economy strategies for managing end-of-life composite blades. While innovations such as the geometric redesign of floating platforms and the aerodynamic refinement of blade components have yielded substantial gains—achieving up to a 30% mass reduction in PLA prototypes—more conservative optimizations of internal geometry configurations in GFRP blades provide only around 7% mass reduction. Nevertheless, persistent challenges related to polymer composite degradation and fatigue under severe weather conditions are driving the adoption of real-time hybrid predictive models. A bibliometric analysis of over 1000 publications confirms more than 25 percent annual growth in research across these interconnected areas. This review serves as a comprehensive reference for engineers and researchers, identifying three strategic frontiers that will shape the future of wind turbine blade technology: advanced composite materials, integrated computational modeling, and scalable recycling solutions.

## 1. Introduction

In a world confronted with global issues such as climate change, the shift toward renewable energy has become a focal point [1,2,3,4]. Wind energy, in particular, is recognized as one of the most viable solutions due to its capacity to produce clean and sustainable electricity. This renewable resource not only aids in decarbonization and enhances global energy security, but it also stimulates local economies by generating employment opportunities in rural and coastal regions [1,3,5]. International bodies, including the United Nations, have underscored the significance of wind energy in achieving the Sustainable Development Goals (SDGs), particularly SDG 7 (affordable and clean energy) and SDG 13 (climate action) [6,7].

The long-term viability of wind energy, a cornerstone of the global transition to renewable power, is critically dependent on the materials used in turbine blades. While polymer composites have enabled the creation of larger and more efficient blades, they also present a significant challenge related to durability, recyclability, and in-service degradation. Issues such as leading-edge erosion (LEE) from atmospheric particles, moisture ingress that compromises structural integrity, and the fundamental difficulty of recycling cross-linked thermoset polymers at their end-of-life are major bottlenecks for the industry [1,2,3,5]. Overcoming these material science hurdles is essential for ensuring the sustainability and economic feasibility of wind power.

Functional and structural analysis of wind turbines is important in order to ensure the reliability, efficiency, and safety of the turbine operation. It helps in analyzing stress and loads acting on the turbines during operation. It aids in proper material selection and designs to meet the required performance. Optimization of the structural and aerodynamic design of wind turbines has been explored using the Isight platform to develop Kriging surrogate models [8]. The optimization process incorporates DAFoam, TACS, Mphys, and SNOPT [9].

Recent studies have explored various aspects of wind turbine improvement: multi-objective blade optimizations achieved moderate gains in torque and mass reduction, reliability analyses have underscored the importance of design standards (e.g., IEC 61400-2 for small turbines), and new composite mega-blades (∼25 MW, 260 m diameter) have been proposed and evaluated for safety [10,11,12,13]. However, challenges remain in scaling designs and validating models against real-world conditions.

The BEM method, utilizing QBlade software, was employed for structural and modal analysis [13]. The energy efficiency of a small wind turbine has been investigated through the study of shape, inclination, and confusor–diffuser casings [14]. The thrust and aerodynamic performance of wind turbine blades have been examined through CFD analysis in conjunction with response surface methodology (RSM), Bi-objective Mesh Adaptive Direct Search (BiMADS), and Reynolds-Averaged Numerical Simulation (RANS) [15]. A strip analysis technique has been introduced for vertical-axis wind turbines to evaluate the 2D shear stress transport (SST) model [16]. A workflow process has been suggested for optimizing a curved bladelet on a wind turbine blade [17]. Using PLA prototypes manufactured by additive processes and optimized through CFD and FEM, the curved bladelet model achieved ~30% higher torque generation while retaining only 70% of its original mass, effectively contributing to a 0.81% increase in the overall blade torque. Previous research has shown more conservative values of about 7% by optimizing the internal configuration of glass fiber polymer composite blades [18].

A wind turbine model was developed to explore the effects of wind speed [19]. The techno-environmental economic assessment of integrating photovoltaic panels with vertical wind turbines has been investigated, using Egypt as a case study [20]. Three-dimensional models were created using SolidWorks. The influence of atmospheric stability on wake flow was utilized to predict wake velocity and turbulence intensity in turbine blades [21].

Recent advancements in wind turbine technology have centered on the aerodynamic and structural optimization of blades and towers, leveraging a combination of computational modeling and experimental validation to enhance power output and efficiency across various turbine architectures [22,23,24,25,26,27,28,29,30,31,32,33]. Simultaneously, a major research thrust involves the development of intelligent control systems, where AI-driven frameworks, adaptive fuzzy logic, and advanced surrogate models are being applied to improve yaw control, blade damping, and the stability of floating platforms [34,35,36,37,38,39,40,41]. Furthermore, to ensure long-term reliability, a third key area focuses on analyzing fatigue and environmental interactions, using dynamic simulations and multiphysics models to understand the effects of extreme weather, wake turbulence, and atmospheric conditions on structural integrity and system resilience [21,42,43,44,45,46,47].

**Figure 1:**
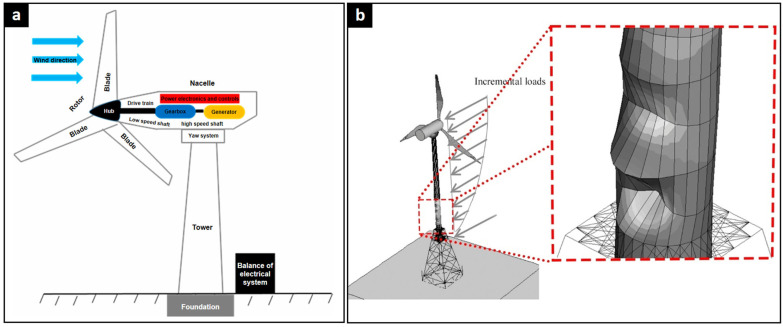
Wind turbine system overview. (**a**) Schematic diagram illustrating the primary physical components of a horizontal-axis wind turbine [48]. (**b**) A finite element model depicting simulated incremental wind loads and the resulting structural deformation at the tower base [49].

Figure 1 presents a general system schematic and a structural simulation model. This dual representation provides valuable information on both the physical configuration and the analytical modeling approaches commonly used in wind turbine design studies [48,49]. The simplified representation of the turbine (Figure 1a) emphasizes its essential parts: the rotor, which includes the blades and hub that capture wind energy; the nacelle, which contains the transmission train with the low-speed shaft, gearbox, high-speed shaft, generator that transforms mechanical energy into electrical energy, and the electronic control and orientation systems. Furthermore, the tower and foundation are depicted, providing structural support, along with the electrical system balance, which signifies the additional elements required for electrical functionality. Conversely, Figure 1b displays a structural analysis model, where incremental loads applied to the rotor are simulated to assess the impact of wind. It also underscores the interaction between the tower and the foundation, utilizing a simulation mesh that facilitates the examination of structural stresses and deformations in the tower resulting from the applied loads. This methodology is commonly employed in wind turbine design and mechanical simulation research [50,51].

Despite numerous studies addressing individual aspects of wind turbine design—such as materials, reliability, and control—there remains a lack of comprehensive reviews that integrate both structural and functional advancements. This work addresses that gap by examining the convergence of aerodynamic design, structural engineering, advanced materials, and intelligent control methodologies that collectively define modern wind turbine performance. With the number of publications in this area growing by approximately 25% annually and over 1500 papers published in 2024 alone, this review responds to increasing research interest by synthesizing recent developments in FEM/CFD modeling, structural health monitoring (SHM), and AI-enabled optimization and maintenance strategies, including innovations in floating turbine systems. Guided by three research questions—(RQ1) identifying dominant methods, materials, and themes; (RQ2) analyzing publication trends over the past five years; and (RQ3) uncovering key research gaps and emerging topics—this review is structured across sections addressing materials, blade optimization, fault detection, offshore design, and environmental performance. A bibliometric analysis performed with details given in Appendix A complements the technical review, offering a holistic perspective that consolidates current knowledge and outlines future directions in the design and sustainable development of next-generation wind turbines.

## 2. Polymer Composite Systems for Wind Turbine Blades

### 2.1. Conventional Polymer Composites and Their Limitations

The selection of materials and structural design strategies plays a critical role in optimizing wind turbine performance. Composite materials, particularly glass and carbon fiber reinforced polymers (GFRPs and CFRPs), are widely adopted due to their exceptional strength-to-weight ratio and ability to withstand cyclic loads [52,53,54,55]. Advanced design technologies such as adaptive aeroelastic tailoring and tow steering have further improved blade stiffness and flexibility, enabling more efficient structural responses under varying wind conditions [55,56,57]. Additionally, innovations like hybrid steel–concrete towers and the use of intelligent materials—such as shape memory alloys—have enhanced the resilience of wind turbine structures against extreme environmental events [58,59]. While hybrid steel–concrete towers may not reduce overall mass compared to all-steel counterparts, they are increasingly adopted for their improved stability, cost optimization, and suitability for modular construction in large-scale wind energy systems.

**Figure 2:**
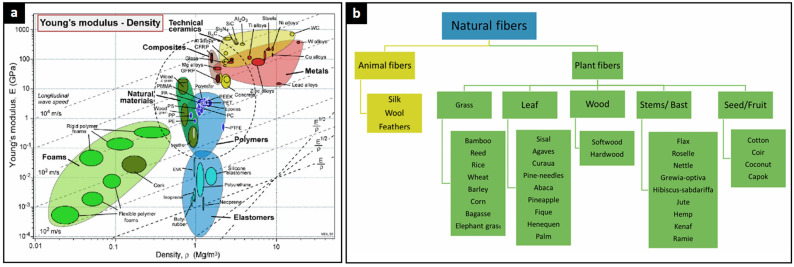
(**a**) Young’s modulus vs. density for materials used in wind turbine components, highlighting the balance between stiffness and weight. (**b**) Classification of natural fibers, with plant-based options showing promise for sustainable composites [48].

As illustrated in Figure 2a, material selection is often guided by the relationship between Young’s modulus (*E*)—a measure of stiffness—and density, both of which are critical for designing lightweight yet structurally effective wind turbine components. While metals and ceramics offer high stiffness, they are generally denser. Composites, on the other hand, combine moderate density with excellent mechanical performance, making them ideal for blade applications. Although stiffness is essential for minimizing deflection under aerodynamic loads, material strength is equally important to ensure resistance to fatigue and extreme loading throughout the turbine’s operational life. Polymers and elastomers provide flexibility and low weight, and foams are commonly used as core materials due to their extremely low density. Recently, natural materials have gained attention for their potential to contribute to sustainable turbine manufacturing.

Figure 2b classifies natural fibers into animal and plant sources, the latter being especially relevant due to their mechanical advantages and renewability. While animal fibers like silk, wool, and feathers have limited structural utility in wind turbines, plant-based fibers—such as flax, jute, hemp, and bamboo—show promise for integration into bio-based composite systems, supporting the transition to more sustainable wind energy technologies. Plant-based natural fibers are increasingly explored as sustainable alternatives to synthetic reinforcements in wind turbine blade manufacturing due to their renewability, biodegradability, and low environmental impact [60]. These fibers are classified by their botanical origin, including grass fibers (e.g., bamboo, cane, and wheat), which are lightweight with moderate strength; leaf fibers (e.g., sisal and fique), known for high tensile strength; and bast fibers (e.g., jute, flax, hemp, and kenaf), which exhibit a favorable balance of stiffness, strength, and flexibility—making them the most promising candidates for structural applications [61,62]. Wood-derived fibers and those from seeds and fruits (e.g., cotton and coconut husks) offer benefits such as low density, good biodegradability, and moisture absorption but are generally limited to non-structural or low-load applications [48].

The integration of natural fibers into turbine blade composites supports circular economy principles and reduces the carbon footprint associated with traditional glass or carbon fiber-reinforced polymers. However, challenges remain due to their lower mechanical performance, higher variability, and sensitivity to moisture, which can affect long-term durability and consistency. Addressing these limitations through hybrid composites, surface treatments, and improved resin compatibility is a growing focus in sustainable wind turbine design.

However, the primary barrier to the widespread adoption of natural fibers in large-scale structural applications is their inherent hydrophilicity. Due to the high percentage of hydroxyl groups in their cellulose and hemicellulose components, these fibers readily absorb moisture from the environment, which significantly degrades their mechanical performance and long-term durability. Recent comprehensive reviews have quantified this degradation, showing that moisture uptake can reduce the tensile strength of natural fiber composites by up to 40% and the elastic modulus by 20–30%, depending on the specific fiber type, its mass fraction in the composite, and the effectiveness of any surface treatments [63,64,65,66,67].

This performance loss is attributed to several coupled mechanisms: swelling of the fibers, which induces internal stresses; plasticization of the polymer matrix; and, most critically, the weakening of the fiber–matrix interfacial bond. This compromised interface reduces load transfer efficiency and accelerates damage mechanisms like microcracking and delamination, which severely impairs the material’s fatigue resistance—the most critical performance criterion for wind turbine blades. This presents a fundamental challenge: the very characteristic that makes natural fibers attractive (their biological origin) is also the source of their greatest weakness in the humid, cyclic-loading environments typical of wind energy applications. While their environmental benefits are clear, the significant compromise in fatigue life due to moisture sensitivity currently limits their application to smaller, less structurally demanding turbines where longevity and extreme load-bearing capacity are less critical. Consequently, a major focus of current research is on mitigating these effects through advanced surface modifications, such as alkaline and silane treatments, and the development of hybrid composites that combine natural fibers with synthetic ones to balance cost, sustainability, and performance [67].

Table 1 presents a comparative overview of the primary reinforcing fibers used in wind turbine blade manufacturing. It is important to note that the mechanical properties of the final composite are highly dependent on factors such as fiber volume fraction, manufacturing process, and fiber orientation; therefore, the values presented represent typical ranges found in the literature. These data reveal a clear performance and cost hierarchy, creating a strategic gap between the cost-effective workhorse material (E-glass) and the premium-performance option (carbon fiber). This gap has driven interest in “middle-ground” materials like basalt and S-glass, as well as hybrid composites, which allow for a more tailored and economically efficient material distribution within the blade structure. For instance, designers can leverage the exceptional stiffness-to-weight ratio of carbon fiber in critical load-bearing sections like the spar cap, while using more economical glass fiber for the less-stressed aerodynamic shells.

**Figure 3:**
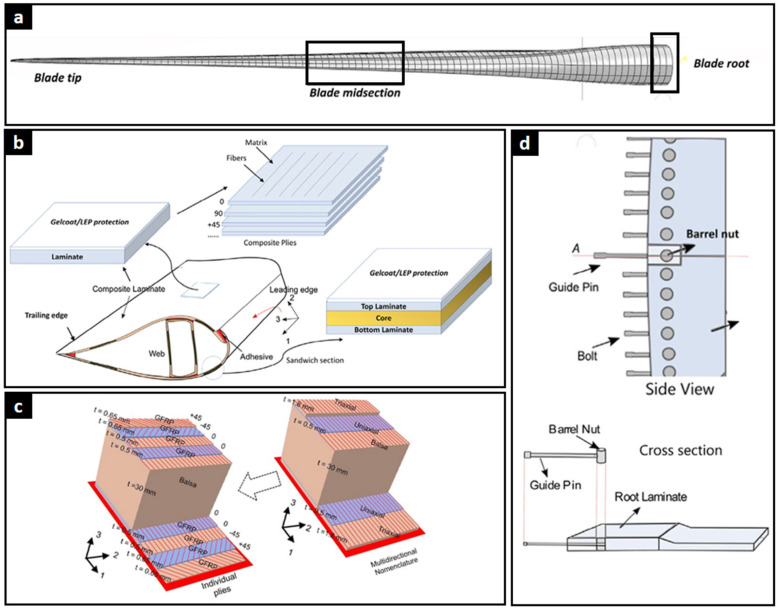
(**a**) Standard segmentation of a wind turbine blade showing the tip, midsection, and root regions. (**b**) Cross-sectional architecture of the blade, including gelcoat/LEP protection, fiber-reinforced composite laminates, adhesive joints, and sandwich structure with a lightweight core. (**c**) Transformation of a multidirectional layup into an individual-ply stacking sequence for detailed structural modeling [73]. (**d**) Blade root connection system incorporating bolts, barrel nuts, and guide pins for secure attachment to the rotor hub [74].

To better understand how these material systems are integrated into blade construction, it is important to examine the internal architecture and structural configuration of wind turbine blades. Figure 3 provides a comprehensive overview of a standard wind turbine blade cross-section, detailing its main structural elements and internal architecture. The blade is typically divided into three key sections: the tip, midsection, and root (Figure 3a). Internally, it consists of multiple layers of fiber-reinforced composite materials, with fibers oriented at 0°, 90°, and ±45°, embedded within a polymer matrix to deliver high strength and stiffness (Figure 3b). A protective gelcoat or leading-edge protection (LEP) layer is applied to the blade surface to mitigate erosion and damage from airborne particles. The cross-section also reveals the use of structural adhesives in critical joints and a sandwich panel configuration, wherein a lightweight core material is encapsulated between composite skins to enhance strength-to-weight efficiency. Accurately defining the composite layup—particularly the individual-ply stacking sequence—is essential for local solid modeling, as it determines the thickness, cohesive interfaces, and material orientation within specific blade regions. In the case of the 10 MW reference blade developed by the Technical University of Denmark (DTU), an iterative procedure was applied to convert an initially multidirectional layup into an equivalent individual-ply sequence, ensuring structural consistency with respect to mass, center of gravity, and sectional geometry (Figure 3c). Finally, Figure 3d illustrates the blade root connection system, including bolts, barrel nuts, and guide components that enable a secure mechanical interface between the blade and the rotor hub.

**Figure 4:**
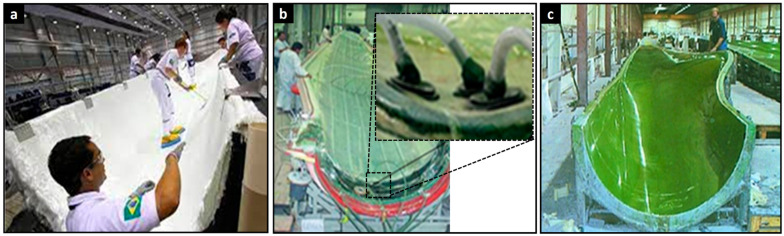
(**a**) Hand lay-up process using manual fiber placement and resin application. (**b**) Vacuum infusion method, ensuring improved resin distribution and reduced defects. (**c**) Vacuum-assisted resin transfer molding (VARTM) technique, providing enhanced process control and final part quality [48].

Building on the structural overview of wind turbine blades, it is essential to understand how the fiber-reinforced polymer composites that form their primary load-bearing components are manufactured. Figure 4 illustrates three primary manufacturing techniques employed in the fabrication of wind turbine blades. In Figure 4a, the hand lay-up process is shown, where layers of fiber reinforcement are manually placed onto a mold and impregnated with resin. This method allows flexibility in design and is cost-effective but highly dependent on operator skill, which can introduce variability. Figure 4b depicts the vacuum infusion process (or use of pre-impregnated fibers), where resin is drawn into the fiber network using vacuum pressure, enabling improved fiber wet-out, reduced void content, and greater consistency compared to manual methods. Finally, Figure 4c shows the vacuum-assisted resin transfer molding (VARTM) technique, a closed-mold process that offers enhanced control over resin flow and curing, resulting in superior part quality and mechanical performance. These manufacturing methods are critical to achieving the desired structural integrity, dimensional precision, and long-term durability required for wind turbine blades operating under complex environmental loads.

### 2.2. Next-Generation High-Performance Composite Materials

Polymer matrix composites remain the most widely used materials in wind turbine manufacturing, though other types—such as metal matrix, ceramic matrix, and carbon matrix composites—are also gaining attention. The key advantage of composites lies in their ability to combine high strength and stiffness with reduced weight, resulting in enhanced mechanical efficiency, lower environmental impact, and improved wear resistance [75,76]. Challenges associated with the use of composites in wind turbine applications have been extensively examined, including damage tolerance, long-term fatigue performance, and end-of-life recyclability [77]. A comprehensive overview of composite systems suitable for wind turbines has been presented in the literature, highlighting their adoption across blade, nacelle, and tower components [71].

The performance of composite blades directly influences the aerodynamic efficiency and energy capture of wind turbines [78]. Accordingly, manufacturing innovations such as pultruded carbon fiber and fiberglass systems have demonstrated reductions in both blade weight and overall cost [79]. However, recycling remains a significant challenge for thermoset-based composites. Multiple studies have addressed the mechanical recycling of composite wind blades and proposed future pathways for improving end-of-life management [80,81].

In contrast to thermoset-based composites, whose end-of-life management poses a significant environmental and economic challenge, thermoplastic composites are gaining favor for their inherent recyclability, weldability, and potential for reduced manufacturing costs [80,82]. A leading example is Arkema’s Elium^®^, a liquid thermoplastic resin compatible with existing infusion processes like VARTM [83], making it a potential “drop-in” replacement for epoxies in current manufacturing facilities. Structural validation studies, including the manufacturing and testing of 13 m blades by the National Renewable Energy Laboratory (NREL), have shown that Elium^®^-based glass fiber composites exhibit stiffness comparable to their epoxy-based counterparts [84]. However, a key performance advantage lies in their superior damping characteristics. The thermoplastic blades demonstrated five to seven times higher structural damping than identical epoxy blades. This enhanced damping can reduce operational loads and vibrations, potentially leading to improved fatigue performance and an extended service life for the entire turbine system. In addition to these damping benefits, thermoplastic blades have demonstrated static and fatigue performance comparable to epoxy [84], with reduced impact damage, as well as higher interlaminar toughness and superior delamination-fatigue resistance, resulting in slower damage growth per cycle [85].

Beyond performance, the economic benefits are substantial. Because Elium^®^ polymerizes at room temperature without the need for a heated post-cure, manufacturing cycle times and capital costs for heated tooling can be significantly reduced [84]. A techno-economic analysis by NREL concluded that these efficiencies could lead to a 4.7% reduction in the final manufactured cost of a blade, even with the currently higher price of the thermoplastic resin [86].

Furthermore, the thermoplastic matrix can be dissolved to recover both the polymer and full-length glass fibers, which retain their mechanical properties, making a closed-loop recycling system economically feasible. This combination of through-life and end-of-life advantages points toward a potential paradigm shift in blade manufacturing and maintenance. The ability to thermally weld thermoplastic components [84] could eliminate adhesive bonds—a primary failure point in current blade designs—and enable the creation of stronger, lighter, more monolithic structures. This also opens the possibility of more effective in-field repairs using localized heating rather than the mechanical grinding required for thermosets, fundamentally altering the lifecycle management of these critical assets. While Elium^®^ is the most prominent commercial example, research into other liquid thermoplastic systems for large-scale infusion continues [87], aiming to further expand the material options for next-generation recyclable blades [88].

To improve performance further, nano-structured composite laminates have been explored, with notable enhancements in fracture toughness and damage resistance [89]. Despite their promise in reducing blade mass, nano-engineered composites face scalability and recycling constraints that must be resolved for commercial viability. Experimental studies on E-glass fiber composites with polyester matrices have demonstrated strong tensile and bending properties [90], while glass fiber reinforced polymers (GFRPs) continue to be favored for their impact resistance and cost-effectiveness. Nevertheless, compared to carbon fiber reinforced polymers (CFRPs), GFRPs exhibit higher density and lower stiffness, which can limit their application in large-scale, high-efficiency turbines.

Efforts to model fatigue behavior in laminated composites have utilized approaches like the Variable Amplitude Miner’s Rule [91] and empirical models that account for fiber volume fraction, ply sequence, stress amplitude, and loading cycles [92]. Simulations and design studies using finite element analysis (FEA) have provided valuable insight into structural performance. For example, a study of flax fiber-reinforced blades designed in CATIA for urban horizontal-axis wind turbines incorporated structural, modal, and harmonic analysis under various load conditions [93], while other studies applied static FEA to conventional blade geometries [62].

Natural fiber composites, such as those incorporating flax, offer a biodegradable and renewable solution for small-scale or urban wind turbines. Hybrid composites combining carbon/glass fibers with silicon carbide (SiC) particles in an epoxy matrix have shown improvements in tensile and impact strength [94]. Vertical-axis wind turbines (VAWTs), commonly used in urban applications, have also benefited from material optimization strategies. A variation of the Discrete Material and Thickness Optimization (DMTO) method has been applied to minimize blade mass while maintaining structural integrity [95]. Additional structural assessments using ANSYS have included evaluations of total deformation, maximum principal stress, Hashin’s failure criterion [96], and strain energy [54], while Campbell diagrams [97] have been used to examine the dynamic behavior of onshore turbines [98]. Despite these advances, composite materials—especially thermoset-based systems—continue to face limitations in recyclability, moisture resistance, and fatigue durability under sustained mechanical and environmental loading. Overcoming these challenges remains essential for advancing next-generation, high-performance, and sustainable wind turbine designs.

### 2.3. Smart and Adaptive Polymer Composite Materials

The pursuit of higher performance and longer lifespan in wind turbine blades has led to growing interest in smart and adaptive structural materials. Among these, shape memory alloys (SMAs) stand out due to their unique ability to undergo deformation at lower temperatures and return to their original shape when heated. SMAs have been proposed as substitutes for conventional spring and dashpot components in tuned mass dampers (TMDs), offering an SMA-based TMD solution to effectively control the seismic response of wind turbine towers [99]. Additionally, embedding SMA layers within composite laminates has shown improvements in adhesion and flexural stiffness [100], while vibration studies of SMA-based blades reveal hysteresis behavior, contributing to structural damping [101].

Another emerging area is the development of morphing structures, which require compliance to minimize actuation force while retaining adequate stiffness for load-bearing. This duality can be addressed through anisotropic composite architectures or materials that exhibit bistable or multistable behavior [102]. While SMAs offer advanced functionalities, their application is limited by high material costs and fatigue behavior influenced by phase transformations. In parallel, self-healing materials, such as aluminum matrix composites, are being studied for their ability to autonomously repair cracks, thereby extending service life and reducing maintenance demands [103].

## 3. Structural and Functional Analysis of Wind Turbines

This section reviews advances in blade design optimization. Beyond material innovations, the computational approaches that enable structural and aerodynamic refinement have advanced significantly.

In modern wind turbine design, Finite Element Method (FEM) and computational fluid dynamics (CFD) are foundational simulation tools. FEM is used for structural analysis, allowing engineers to model and predict stresses, deformations, and vibration modes, and damage [104,105,106] in solid components like blades and towers under various static and dynamic loads. CFD, conversely, is used for aerodynamic analysis, simulating airflow patterns [107,108], pressure distributions, and aerodynamic forces on the turbine (Table 2). For instance, A CFD-based study optimized the S809 airfoil geometry of the NREL Phase VI turbine blade to achieve variable-speed performance without pitch control, reducing system complexity and cost [109].

For a complete picture of aeroelastic behavior—how the flexible structure and the moving fluid influence each other—these methods are coupled in what is known as fluid–structure interaction (FSI). FSI simulations provide a high-fidelity understanding of how blades behave under dynamic conditions like gusts, turbulence, and rapid control actions [52,56,110,111].

While high-fidelity models are essential, faster tools are needed for initial design loops. Aerodynamic models like the Blade Element Momentum Theory (BEMT) provide rapid performance predictions, allowing for the optimization of key parameters like the power coefficient (Cp) and tip speed ratio (TSR). These models are integrated into aero-servo-elastic simulations, which combine aerodynamics, structural dynamics, and control system responses. These integrated simulations are crucial for designing and testing control strategies, such as individual and collective pitch control, to maximize energy capture while minimizing structural loads across the turbine’s operating range [112,113,114].

In the design process, a hierarchy of models is used. Low-fidelity aerodynamic models like BEMT offer high speed for initial optimization, while high-fidelity CFD models provide the accuracy needed for detailed load prediction and refinement. The choice depends on the design stage and available computational resources.

To validate these models and ensure long-term reliability, real-time monitoring is critical. Structural health monitoring (SHM) systems, often using fiber optic sensors, track a blade’s structural response (e.g., strain and vibrations) in real time. These data, along with operational data from SCADA systems, are increasingly analyzed with artificial intelligence to detect damage, predict component failure, and optimize maintenance schedules, thereby reducing operational costs and improving turbine reliability [115,116,117,118].

### 3.1. Optimization of Polymer Composite Blade Design

The core of modern blade design is Multidisciplinary Design Optimization (MDO), a process that uses powerful algorithms to find the best possible design by balancing competing objectives. However, computational tools alone are insufficient. Experimental tests, including wind tunnel assessments and free-vibration evaluations, remain essential for validating the numerical models used in MDO, especially for complex phenomena like wake formation and structural fatigue [119,120,121].

**Figure 5:**
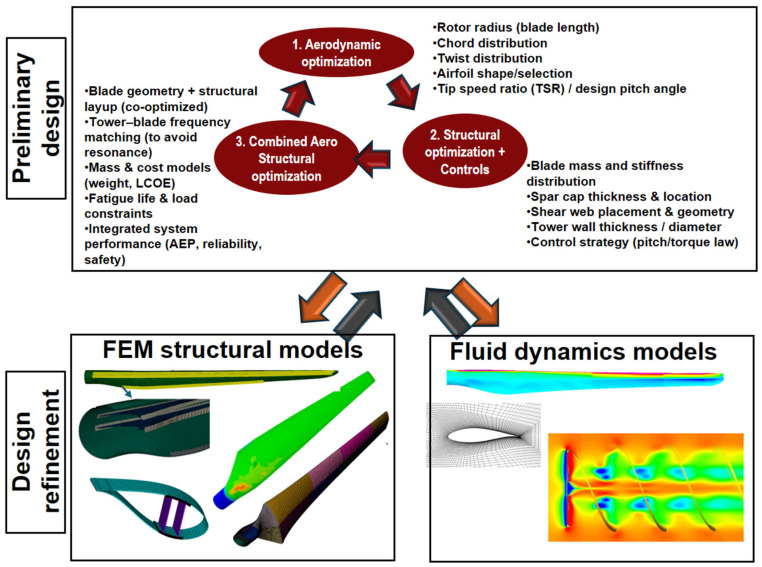
Multidisciplinary process for structural and aerodynamic optimization and refinement in wind turbine design [122].

MDO frameworks use optimization algorithms and metaheuristic techniques to create innovative and efficient designs, while ensuring adherence to international safety and performance standards, such as those from the IEC [122,123]. A typical MDO workflow is illustrated in Figure 5.

The MDO process is an iterative loop. It typically begins with aerodynamic optimization, where parameters like chord and twist are adjusted to maximize Annual Energy Production (AEP). This is followed by structural and control optimization, where the blade’s internal structure is refined to minimize mass and cost. This entire design loop is then checked and refined using high-fidelity 3D structural (FEM) and aerodynamic (CFD) models. This allows engineers to analyze critical details like buckling, fatigue, and complex 3D airflow, ensuring the final design is a robust balance of performance, cost, and structural reliability.

### 3.2. Aerodynamic and Structural Analysis of Composite Blades

Recent research focuses on integrating novel aerodynamic features directly with structural considerations. One area of focus is biomimicry, with studies showing that eagle-inspired airfoils or leading-edge protuberances can improve aerodynamic performance [124,125]. Another major research thrust is the computational optimization of blade geometry. Various methods are employed, from response surface methodology (RSM) for optimizing chord and twist angle [126,127] to advanced approaches using Artificial Neural Networks (ANNs) [128] or genetic algorithms to optimize ply lay-up patterns [129]. Algorithms like the Artificial Bee Colony (ABC), grounded in BEM theory, have also been developed to enhance aerodynamic performance [130]. While these computational methods often report lighter or more efficient blade designs, challenges in scalability and full-scale experimental validation remain. Research has also extended to specialized designs, such as Archimedes Spiral Wind Turbines for urban environments [131].

### 3.3. Coupled Fluid–Structure Interaction Models for Composite Blade Analysis

To ensure structural integrity, an accurate understanding of fluid–structure interaction (FSI) is essential. Two-way coupled FSI models are used to assess fatigue loads by accounting for the complex interplay between inflow conditions and the flexibility of the blade and tower [132]. Such simulations, often combining CFD with FEM software like ANSYS, allow engineers to identify critical stress concentrations and tip deflections under various operating conditions [133,134]. FSI modeling is critical for analyzing both new and damaged blades, as phenomena like cracks and erosion can alter aeroelastic behavior [135]. Studies have shown that blade tip modifications can enhance power generation while maintaining structural integrity [136], and FSI is vital for analyzing novel concepts like segmented blades [137]. While computationally intensive, FSI models are particularly necessary for accurately representing turbine performance in cases involving high strain and blade flexibility [138], and they provide deeper insight into how blade flexibility influences the turbulent wake [139]. Although BEM-based approaches can be used for FSI [140], CFD-based coupling remains the high-fidelity standard, and improving its efficiency and accuracy remains an active area of research [141,142].

## 4. Monitoring and Fault Detection

This section discusses advanced solutions for wind turbine monitoring, such as structural health monitoring (SHM) of composite blades, including AI and IoT applications of blades and supporting structures.

### 4.1. Structural Health Monitoring (SHM) of Composites Blades

Despite technological advancements, wind turbines face persistent challenges from structural fatigue, wind-induced vibrations, and unpredictable dynamic loads. While prior studies have leveraged simulations and wind tunnel testing to improve component design [52,74,110,119], real-world operational monitoring is crucial. Structural health monitoring (SHM) systems, often coupled with control algorithms, are effective tools for prolonging the operational lifespan of turbines [58,116,138,143].

**Figure 6:**
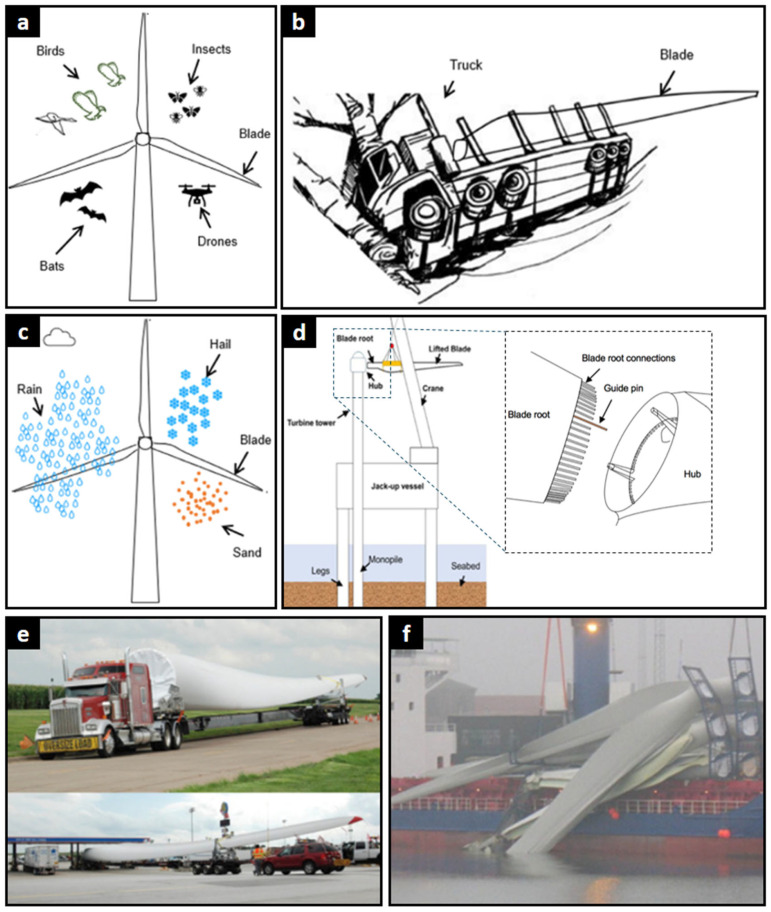
Risks and damage in the transport, operation, and assembly of wind turbines [74,144]. (**a**) and (**c**) highlight operational risks, including collisions with wildlife like birds and bats, as well as drones, and environmental damage from weather phenomena such as rain, hail, and sand erosion. (**b**,**e**,**f**) shows potential damage to blades during road accidents and logistical challenges during overland and sea shipping. (**d**) depicts a critical assembly risk: the potential for a collision between the blade root and the hub during the complex, crane-assisted offshore installation process.

Figure 6 shows various failure risks that necessitate robust SHM systems for early damage detection. These include in-operation collisions with wildlife and drones (a); damage during land transport (b, e); blade erosion from environmental factors like rain, hail, and sand (c); and the risk of collision during offshore installation (d, f). All these risks can potentially damage blades and could significantly affect the structural integrity and operation compared to the design prototypes.

### 4.2. AI and IoT Applications for Composite Health Monitoring

Advanced monitoring methods have greatly enhanced turbine reliability. Fiber optic sensors with Bragg gratings can detect microcracks, vibrations, and deformations in real time at critical locations like the blade root and tower [116]. Data from these sensors can feed predictive maintenance software, potentially reducing downtime by up to 30%.

**Figure 7:**
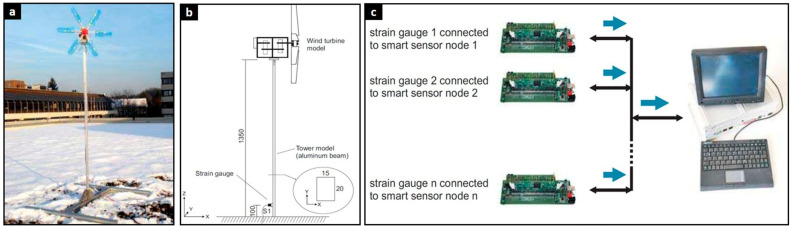
Experimental setup: (**a**) A model of a wind turbine was exposed to actual environmental excitations by wind loads on the top of a building. (**b**) Strain gauges were mounted nearly at the bottom of the beam. (**c**) Load monitoring system based on decentralized preprocessing with smart sensor nodes [116].

Figure 7 shows an experimental setup for a load monitoring system that uses decentralized preprocessing with intelligent sensor nodes [116]. Figure 7a,b show the experimental model: a photo of the small-scale turbine installed outdoors (a) and a schematic detailing the tower dimensions and strain gauge placement near the base (b). This setup provides experimental data on the turbine’s structural behavior under real-world loads. The right panel (c) shows the system architecture, where strain gauges connect to smart sensor nodes. These nodes process data locally before transmitting it to a central system, improving real-time monitoring efficiency.

Beyond SHM, computational tools remain vital. Methodologies for multidisciplinary optimization using FEM have been shown to reduce blade weight by 15% without sacrificing integrity [52]. Similarly, CFD simulations using RANS models have been used to better understand wake formation and reduce turbulence impacts by 20% through blade pitch control modifications [110]. For offshore turbines, historical weather data can be used to simulate extreme wind and wave loads, allowing for the development of active control strategies that mitigate the effects of unforeseen gusts [145]. The adoption of IoT technologies and SCADA systems has transformed wind farm management. Real-time monitoring platforms that use cloud communication can oversee temperature, mechanical load, and generator efficiency [118]. Advanced control strategies leveraging these systems can reduce failure response times by 25%.

**Figure 8:**
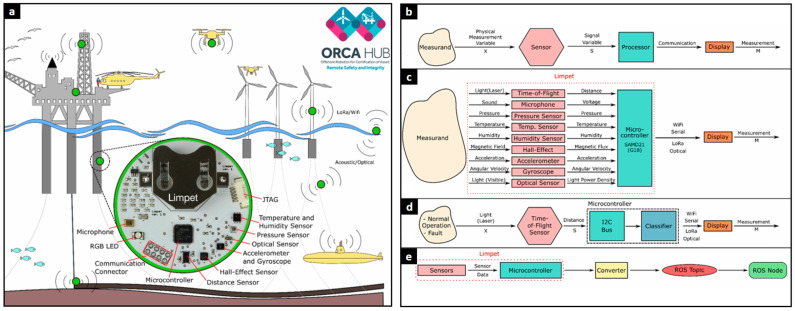
The ORCA Hub’s Limpet sensor system: (**a**) Deployment concept in an offshore environment. (**b**) General instrument model. (**c**) Limpet multi-sensor architecture. (**d**) Fault detection logic. (**e**) Integration with Robot Operating System (ROS) [118].

Figure 8 details the Limpet, a multi-modal IoT sensor platform developed for this purpose. The figure illustrates the Limpet sensor system within the ORCA Hub, a remote monitoring framework for offshore infrastructure. Figure 8a provides a conceptual overview of the deployment scenario. The hardware, shown in the inset, integrates a suite of sensors including microphones, accelerometers, and optical sensors. Figure 8b shows the general instrument model, while Figure 8c details the Limpet’s multi-modal sensing architecture handled by its microcontroller. A specific fault detection use case is shown in Figure 8d, and its integration with the Robot Operating System (ROS) is depicted in Figure 8e, enabling sensor data to be converted into ROS topics for advanced robotic and IoT monitoring.

### 4.3. Structural Optimization of Supporting Structures

While blades are often the focus, the tower is the backbone of a wind turbine, and its design presents a significant opportunity for cost reduction and performance enhancement. Modern optimization techniques are moving beyond simple steel tubes to create smarter, more resource-efficient structures.

A prime example is the optimization of hybrid steel–concrete towers, particularly for turbines with hub heights exceeding 100 m, where traditional steel towers become prohibitively expensive and difficult to transport. Research by Chen et al. [146] demonstrates the use of evolutionary algorithms to optimize the material distribution in these hybrid towers. By assessing performance against both wind and seismic loads, their simulations showed that optimized designs could reduce manufacturing costs by 18% and diminish structural oscillations by 12%, creating a more resilient and economical structure for taller turbines.

**Figure 9:**
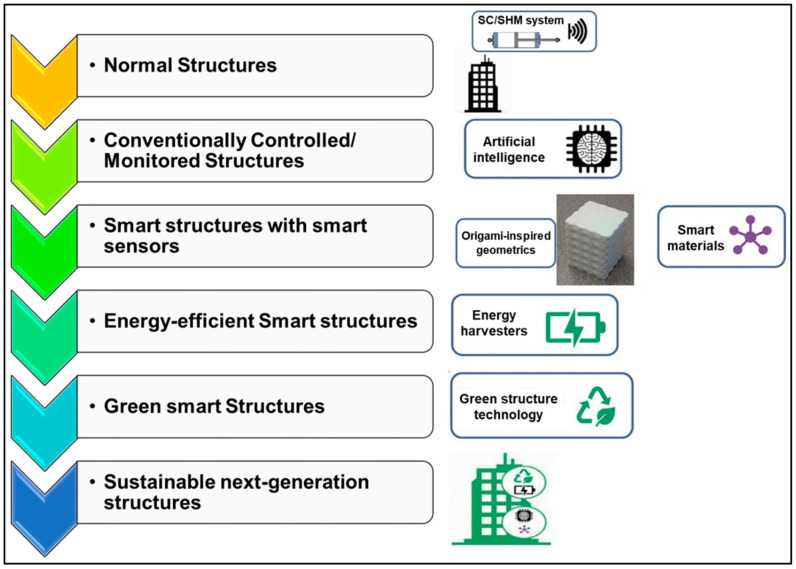
Technological integration for the sustainable next-generation wind energy towers [147].

Another approach gaining traction is the revitalization of lattice tower designs using advanced AI. Historically common for smaller turbines, lattice structures are highly material-efficient but complex to design. A recent comprehensive review highlights the use of AI-driven tools to navigate this complexity [36]. These algorithms can optimize the geometry of thousands of individual elements to minimize weight and cost while meeting strict strength and fatigue criteria. This AI-assisted approach makes lattice towers a viable and competitive alternative for next-generation, large-scale onshore projects. These case studies illustrate a clear trend: the integration of advanced computational modeling and AI is enabling a new generation of wind turbine designs. By moving beyond monolithic approaches and embracing material hybridization and complex geometries, engineers can create towers that are not only taller and more resilient but also more economically and environmentally sustainable (Figure 9).

The integration of IoT and SHM systems in monitoring and fault detection of wind turbine composite blades represents a significant leap forward. However, current architectures face critical limitations that must be addressed to unlock their full potential. Two of the most pressing challenges are power consumption and data management, which include latency and bandwidth issues. Many wireless sensors are deployed in remote or difficult-to-access locations, such as blade tips or offshore platforms. This makes battery replacement impractical and costly, rendering the power supply a primary bottleneck for long-term, autonomous monitoring. The continuous operation of a dense network of high-fidelity sensors and communication modules leads to energy demands that current battery technology cannot sustainably meet. Furthermore, the “data deluge” from these sensor networks creates significant challenges in transmission and processing. The high volume and velocity of structural response data can lead to network congestion and data latency, undermining the effectiveness of real-time control and early fault detection. Transmitting vast quantities of raw data to a central cloud server is often inefficient and, for applications requiring immediate action—such as adaptive blade pitch control in response to a sudden gust—the induced latency is unacceptable.

To overcome power and data limitations in wind turbine monitoring, future systems are integrating energy harvesting for self-sustaining sensors and edge computing to process data locally. Subsequently, on-chip AI fuses sensor data to generate rich diagnostics, which are then transmitted efficiently over long-range, low-power networks. Future research is actively focused on developing next-generation IoT/SHM systems that are more autonomous, efficient, and intelligent.

## 5. Case Studies in the Analysis of Polymer Composite Blades

This section analyzes a selection of key studies that exemplify the advanced analysis, optimization, and monitoring techniques for polymer composite blades discussed previously. Table 3 provides a comprehensive summary of these publications, which are categorized by research area and detailed below to illustrate the breadth of current innovation in composite blade technology.

A clear trend in composite blade analysis is the use of computational algorithms to achieve quantifiable performance gains. Studies show genetic algorithms increasing power output by 10% [28] and specialized HBC-COMEA algorithms boosting torque by over 5% [32]. These computational approaches are validated through rigorous physical and simulated tests, including wind tunnel experiments that confirm design accuracy to within 2% error [27]. The focus extends to entire systems, with active load control strategies reducing fatigue and capital costs in upscaled rotors [26] and CFD models guiding the design of components like endplates for vertical-axis turbines [29]. This optimization mindset is even being applied to different turbine types, including Concentrator Augmented Wind Turbines (CAWTs), where new hardware can double the power coefficient [33]. This hardware innovation is matched by advances in control strategies and smart systems. Here, the integration of artificial intelligence is enabling more scalable and sustainable designs [36]. At the farm level, dual yaw control strategies have been shown to increase overall power output by more than 11% [35]. At the turbine level, multi-objective adaptive fuzzy control creates more robust and dynamic performance across operating conditions [30], while other models focus specifically on using pitch control for blade damping [38]. For the unique challenges of floating offshore wind turbines (FOWTs), researchers are developing real-time controllers using neural networks and super-twisting algorithms to improve stability in harsh sea conditions [41]. Underpinning these designs is a deep focus on fatigue, vibration, and structural analysis. Advanced finite element models are crucial for predicting the compression-bending capacity of concrete towers with minimal error [25] and for designing economically efficient UHPC–steel hybrid towers [31].

Beyond static strength, dynamic analysis is key. Researchers use spectral analysis to understand and mitigate vibrations through blade-structure coupling [47] and implement novel hardware like HSFD systems to directly reduce fatigue damage [44]. These analyses also encompass complex multiphysics challenges, from modeling the electrical impedance of the drivetrain to ensure stability [39] to simulating the dominant structural loads induced by typhoons [46]. Finally, these analyses are increasingly informed by sophisticated environmental and simulation models. High-fidelity Large Eddy Simulations (LESs) are used to accurately model the effects of atmospheric stability on turbine performance [21], while 6-DOF dynamic models can capture how a FOWT’s own pitch motion increases turbulence in its wake [43]. To predict the impact of extreme weather, complex coupled models like WRF-SWAN-FVCOM are used to identify critical instabilities caused by combined wind–wave forces [42]. A key finding across many studies is the importance of selecting the right tool, noting that high-fidelity CFD (RANS) is significantly more accurate than faster BEM-based tools for analyzing performance in post-rated wind speeds [148]. Collectively, these studies demonstrate a clear shift toward a systems-level, data-driven approach. The convergence of AI-powered optimization, high-fidelity multiphysics simulation, and advanced control theory is enabling the design of wind turbines that are not only more powerful but also more resilient, cost-effective, and adaptable to their environment.

## 6. Offshore Wind Turbines

Beyond the monitoring systems discussed previously, offshore wind turbines face a unique and severe set of challenges—spanning operational costs, environmental loads, and logistical complexity—that are driving significant innovation in their design and simulation.

### 6.1. Operational and Economic Challenges

**Figure 10:**
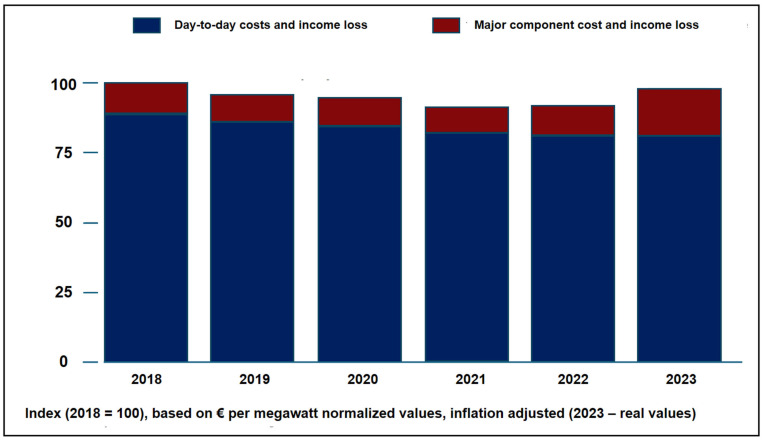
Offshore wind industry’s average costs and lost revenue for operations and maintenance in Europe [149].

A primary challenge facing the offshore wind industry is mounting operational cost pressure, which has reversed a multi-year trend of cost reduction. As illustrated in Figure 10, after several years of declining expenses, the average inflation-adjusted costs and lost revenue for operations and maintenance (O&M) in the European sector increased by 7% in 2023. The data clearly indicate that this surge is not from day-to-day activities but is driven by the escalating costs associated with major component failures and replacements. This financial pressure underscores the urgent need for the advanced design, monitoring, and simulation tools discussed in the following sections, as operators seek to improve reliability and control costs [149].

### 6.2. Environmental Loads and Installation Challenges

Offshore environments present significantly more demanding conditions than onshore sites, requiring wind turbine foundations to be designed for deep waters and variable seabed conditions, which complicates geotechnical analysis and drives innovation in foundation and mooring solutions. Turbine structures must also withstand extreme environmental loads, including high wind speeds and large waves, necessitating robust structural resilience [150].

Recent advancements in environmental load modeling have improved understanding of how turbines respond to complex offshore conditions. Jian et al. [151] conducted a detailed structural assessment of a mega offshore wind turbine tower using finite element analysis, modeling wind loads on the blades and tower separately. Under extreme conditions—such as a 25 m/s wind speed at 10 m height—the wind load on the rotor was calculated to be approximately 10,094 kN, applied at the rotor hub. Tower wind loads were modeled using height-dependent wind pressure distributions and integrated over the tower’s surface to capture variations in air density, wind shear, and shape effects. These analyses underline the importance of accurate environmental load representation during design. Additionally, the tower was evaluated under multiple load cases, including system weight, rotor aerodynamic loads, and tower drag forces, with constraints simulating fixed boundary conditions at the tower base. The findings emphasize that the resilience of offshore wind towers depends not only on structural form but also on precise modeling of wind and wave interactions across operational and extreme states.

Furthermore, offshore wind farm installations also interact with marine ecosystems, raising ecological concerns such as impacts on bird and bat migration [152]. These environmental and installation challenges underscore the need for integrated design approaches that address structural, operational, and ecological resilience simultaneously.

### 6.3. Innovations in Supporting Platforms for Offshore Turbines

To unlock the immense wind resources in deep water where fixed-bottom foundations are not feasible, the industry is rapidly advancing floating offshore wind turbines (FOWTs). This shift has led to a wave of innovation in platform design. Radical new concepts are emerging, such as the “SeaTwirl” turbine, a vertical-axis design that uses the counter-moment of a deep keel for stability. In a Chilean case study, a tri-floater semi-submersible for a 5 MW turbine achieved a steel mass of 1172 t versus 1900 t and 1582 t in comparable concepts—reductions of ~39% and ~26%, respectively, and >30% versus other proposed models—implying substantial material and installation cost savings (reduced raw steel and crane capacity). Under an extreme 13 m wave case, finite-element checks met DNV rules, with a cover safety factor of 1.39 at 294 kPa, external-plate maximum stress of 230 MPa with 3.26 mm displacement (S275 steel), and a buckling factor of safety of 6.63, indicating robust reliability margins [153]. Other innovations focus on hybrid systems, integrating devices like Oscillating Water Columns (OWCs) into floating platforms to actively damp wave-induced pitch and yaw motions [154]. Designing and optimizing these complex floating systems requires immense computational power. Researchers are applying a range of optimization techniques, from classical algorithms to advanced multi-objective evolutionary methods. Recent breakthroughs include using deep neural networks combined with genetic algorithms (NSGA-III) to design mooring systems that significantly reduce platform motion and tension loads [155], and applying Kriging–BAT algorithms for multidisciplinary optimization that successfully reduces the overall levelized cost of energy [156].

### 6.4. Advanced Simulation Tools for Offshore Systems

The complexity of FOWTs demands a new generation of sophisticated simulation tools capable of modeling coupled physical domains. A spectrum of aerodynamic models is used, from the computationally fast Blade Element Momentum (BEM) theory for initial design loops to high-fidelity computational fluid dynamics (CFD) for detailed, blade-resolved analysis of complex flow conditions [157]. In practice, BEM remains a fast, credible baseline for blade load prediction under largely attached flow—when supplied with high-quality polars (preferably 3D/CFD-derived) and careful interpolation, it can match CFD sectional loads across the span even at higher wind speeds [158]. Under turbulent inflow, however, BEM reproduces mean forces mid-span but overestimates variability: versus blade-resolved CFD, equivalent fatigue loads differed by up to 16% (torque) and ~29% (thrust), with strong PSD deviations beyond ~1.5 Hz [159]. Consequently, for fatigue-relevant spectra, root/tip 3D effects, and yaw/separation, blade-resolved CFD is the more reliable load benchmark, while BEM is best reserved for rapid design loops and long-horizon case sweeps [159]. While BEM (or BEM+wake/FVM) is efficient, it cannot resolve detailed velocity fields around the rotor the way CFD can [160]. Finally, modified BEM formulations and data-assisted corrections are emerging in narrow gaps (e.g., improved yaw modeling) but still rely on CFD/experimental data for calibration [161].

A key trend is the development of open-source and modular simulation frameworks. A novel Python-based tool, for example, was recently developed to offer flexible modeling of FOWTs [162], while other codes like WindSE are built for high-dimensional sensitivity analysis [163]. The most powerful approaches involve coupling multiple specialized tools. For instance, a state-of-the-art simulation was achieved by integrating OpenFAST (for structural dynamics), OpenFOAM (for CFD), and MoorDyn (for mooring analysis) to create a comprehensive model that accurately captures the tightly coupled servo-aero-hydro-elastic behavior of an FOWT under realistic conditions [164]. These advanced simulation capabilities are critical for reducing design risk, automating installation planning [157], and accelerating the deployment of next-generation offshore technology.

## 7. Resilience of Wind Turbine Polymer Composites to Extreme Weather Events

Wind turbines are engineered to endure severe weather, yet they remain vulnerable to damage from events that exceed design specifications, such as extreme cyclones and tornadoes. Analyzing site-specific weather is critical; for example, statistical analysis of wind gusts in the UK revealed that some regions are less favorable for offshore development due to the high frequency of extreme events [165]. Such events directly impact a turbine’s lifespan and structural integrity. High-intensity cyclones can significantly shorten fatigue life [166], while operational errors during storms, such as yaw misalignments, can lead to increased vibrations and blade deformations [167]. On a component level, analyses show that extreme typhoon winds induce significant stress concentrations in the gearbox and generator mounts, highlighting them as key failure points during such events [168].

In response to these vulnerabilities, the engineering focus has shifted toward designing for resilience—the ability of a wind energy system to anticipate, absorb, adapt to, and recover from disturbances. To put this concept into practice, researchers are developing advanced resilience frameworks. One approach defines four key capabilities—responding, monitoring, anticipating, and learning—as essential for managing offshore systems [169], while another employs a hierarchical system-of-systems model for comprehensive asset integrity management [170]. The benefits of these frameworks are tangible, with case studies demonstrating that improved resilience planning can significantly reduce restoration time, increase safety, and cut economic losses following a major disruption [171]. Resilience strategies are being applied at multiple scales, from analyzing the impact of random events on a single turbine using Monte Carlo simulations [172] to evaluating the stability of the entire power grid under high wind penetration [173]. This holistic approach extends across the turbine’s full lifecycle, prompting new strategies for the decommissioning and repowering of offshore infrastructure at its end-of-life to maintain system integrity [174].

## 8. Research Frontiers in Polymer Composite Wind Turbine Technology

**Figure A1:**
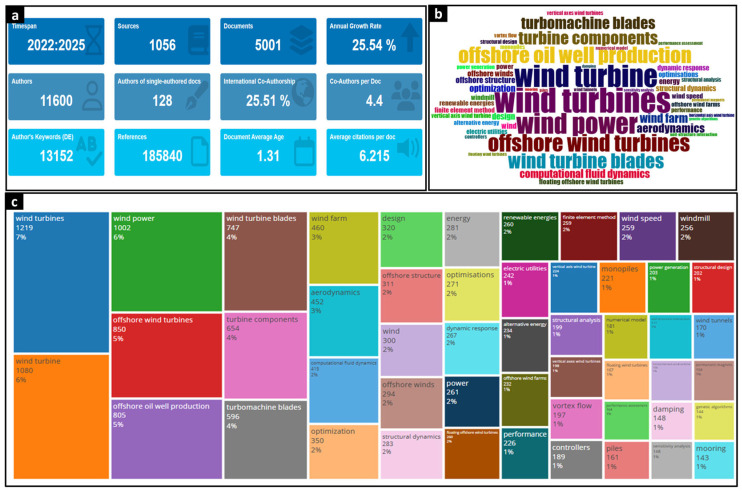
Bibliometric overview of the dataset (2022–2025). (**a**) Main information dashboard summarizing the 1056 analyzed documents. (**b**) Word cloud highlighting the most frequent terms. (**c**) Treemap providing a quantitative breakdown of keyword prominence.

A bibliometric analysis of 2226 publications from 2020 to 2025 confirms a clear shift in research focus. As shown in the keyword analysis (Figure A1, Appendix A), emerging topics like “hybrid models”, “digital twins”, and “offshore wind” are seeing significant growth. This indicates a move toward data-driven, systems-level engineering. While foundational topics like “blade design” remain central, there is a pressing need to address the challenges and research gaps revealed by these new frontiers. The following sections explore the three most critical frontiers shaping the future of wind turbine technology.

### 8.1. Hybrid Modeling and Intelligent Control Systems for Composite Structures

The primary frontier in turbine simulation and control is the move away from purely physics-based models toward hybrid models that integrate real-world data. These models are essential for creating high-fidelity digital twins—virtual replicas of operational turbines used for diagnostics and validation [175]. Current research demonstrates a wide range of applications, from using hybrid Unsteady Reynolds-Averaged Navier–Stokes (URANS) and RANS–Large Eddy Simulation (LES) models for detailed aerodynamic analysis [176] to employing Long Short-Term Memory (LSTM) networks to capture the dynamic characteristics of wind turbines from operational data [177]. This integration of physical principles with data-driven architectures allows for more accurate power curve modeling [178] and virtual prototyping under realistic conditions [179]. However, significant gaps remain. The most pressing challenge is the integration of high-fidelity climate predictions with real-time adaptive control systems to improve resilience in extreme weather [180]. While advanced control actuators, such as those using “fishing line” artificial muscles for mooring stabilization, are being developed [181], fully integrating them with predictive data from SCADA/IoT systems remains a major hurdle [117]. Closing this gap is essential for developing turbines that can not only predict but also dynamically respond to their environment.

### 8.2. Advanced Polymer Composites and the Search for Sustainability

The second major frontier lies in materials science, driven by the dual goals of enhancing performance and ensuring sustainability. Research continues to focus on developing composites with a higher stiffness-to-weight ratio and greater fatigue resistance. While carbon and glass fiber composites remain the standard, significant interest is growing in alternatives. Basalt fibers, for instance, are noted to be up to 30% stronger and 15–20% stiffer than E-glass, while being cheaper than carbon fiber [71]. Furthermore, advanced materials like thermoplastic resins (e.g., Elium^®^) are being explored as replacements for traditional thermoset epoxies, as they offer easier repair and, crucially, recyclability. Despite these innovations, the practical application of advanced materials faces persistent challenges. While studies demonstrate the benefits of novel aeroelastic blade designs [56], there is a lack of research on the long-term durability, manufacturing scalability, and cost-effectiveness of these new materials in real-world operating conditions. Further investigation into the use of natural fibers (e.g., flax and hemp) and bio-based composites is also needed to minimize the lifecycle carbon footprint of turbine blades.

### 8.3. The Circular Economy Imperative

As the first generation of wind farms reaches the end of its 20- to 25-year lifespan, the industry faces an urgent challenge: the end-of-life management of turbine components [182]. This has established the circular economy as a critical research frontier. The primary difficulty lies in recycling the thermoset composite materials used in most blades, which are difficult to break down. Current research focuses on several pathways. An overview of recycling technologies highlights methods like pyrolysis (thermal decomposition) and mechanical grinding for reuse as filler material [183,184,185]. However, these methods can be energy-intensive or result in significant downcycling of the material [186]. A more promising approach is the repurposing of decommissioned blades in architectural or civil engineering applications, such as pedestrian bridges, with studies showing potential material cost reductions of over 70% [187,188]. To accelerate progress, industry-led initiatives are working to establish standards for decommissioning, but scalable, economically viable solutions for blade recycling are still in the early stages of development.

## 9. Conclusions

This review confirms that wind turbine technology is at a pivotal juncture, driven by the dual imperatives of enhancing energy capture in more challenging environments and ensuring long-term environmental sustainability. The research landscape is rapidly evolving from a focus on optimizing individual components to a holistic, systems-level approach that integrates advanced materials, intelligent monitoring, and lifecycle management. The key conclusions of this review are summarized as follows:

- The optimization of wind turbines is no longer a sequential process. Integrated MDO frameworks that combine aerodynamics, structural mechanics, and control systems are now standard, enabling the design of lighter, more efficient, and more reliable systems.
- The integration of AI, IoT, and advanced sensors is transforming turbine operation. From SHM systems that detect damage in real-time to hybrid digital twins that predict failures, data-driven approaches are becoming essential for improving performance and reducing operational costs.
- The push into deeper, more remote waters is the primary catalyst for radical innovations in floating platforms, mooring systems, and advanced FSI simulation tools. These developments are essential for unlocking the vast potential of offshore wind. Resilience is a new design pillar. As turbines face more extreme weather events, designing for resilience has become as important as designing for efficiency. This requires a shift toward system-level thinking, probabilistic risk assessment, and the integration of real-time weather forecasting with adaptive turbine controls.
- Sustainability is an urgent, unsolved challenge. While advanced materials continue to improve performance, the wind industry faces a critical challenge in managing blades at their end-of-life. The development of scalable recycling technologies and circular economy business models is no longer optional but essential for the long-term viability of the industry.

To address these challenges, future research must focus on advancing hybrid modeling capabilities that fuse real-world data with predictive simulations and creating truly adaptive control systems that allow turbines to respond intelligently to severe weather. Furthermore, a dedicated roadmap for integrating circular economy strategies into blade end-of-life management is essential. This roadmap should prioritize three research directions: first, accelerating the development and validation of next-generation materials designed for circularity, such as thermoplastic composites that permit closed-loop recycling; second, improving the economic viability of recycling technologies like pyrolysis to recover high-value fibers from the legacy fleet of thermoset blades; and third, establishing design standards and validation pathways for high-value repurposing of decommissioned blades in civil infrastructure. Success in these areas, supported by updated international design standards, will be crucial for ensuring that wind energy can meet its promise as a cornerstone of the global transition to a sustainable energy future.

## Figures and Tables

**Table 1:** Comparative mechanical properties of reinforcing fibers for wind turbine composites [68,69,70,71,72].

Material	Tensile Strength (MPa)	Young’s Modulus (GPa)	Density (g/cm^3^)	Key Advantages	Key Limitations
E-Glass Fiber	2000–3800	72–85	2.54–2.70	Low cost, good insulation, corrosion resistance	Lower stiffness, higher density than carbon fiber
S-Glass Fiber	4590–4832	88–91	2.46–2.49	~40–50% higher strength and ~10–20% higher stiffness than E-glass	Higher cost than E-glass
Carbon Fiber (Standard Modulus)	3500–5000	230–240	1.8	Excellent stiffness-to-weight ratio, superior fatigue life	High cost, lower damage tolerance than glass fiber
Aramid Fiber (e.g., Kevlar^®^ 49)	~3000	110–130	1.44	Excellent impact resistance, high tensile strength-to-weight ratio	Poor compressive strength, difficult to machine, high cost
Basalt Fiber	3000–4840	85–95	2.65–3.00	Superior to E-glass in strength and stiffness, good thermal/chemical resistance	Higher cost than E-glass, less mature supply chain
Natural Fibers (Flax, Hemp)	500–1500	50–70	1.5	Low cost, low density, biodegradable, good damping	Low mechanical properties, high moisture absorption, property variability

**Table 2:** Simulation tools for structural and functional analysis of wind turbine design.

Parameter	Finite Element Method (FEM)	Computational Fluid Dynamics (CFD)
Primary Domain	Solid Mechanics	Fluid Dynamics
Governing Equations	Equations of solid mechanics and elasticity	Navier–Stokes equations
Key Outputs	Stress, strain, deformation, natural frequencies, buckling loads	Pressure, velocity, lift and drag forces, wake characteristics, turbulence
Computational Cost	High, dependent on mesh density and non-linearity	Very high, especially for turbulent, unsteady flows (e.g., DES, LES)
Primary Use in Blade Design	Structural integrity analysis; vibration and modal analysis; fatigue life prediction; material failure simulation	Airfoil and blade aerodynamic performance; predicting aerodynamic loads; wake and turbine interaction analysis; stall and flow separation studies

**Table 3:** Synthesis of recent research in wind turbine design, analysis, and optimization.

Area	Study Focus	Ref.	Methodology	Key Findings
Aerodynamic and Structural Design	Blade aerodynamic design	[27]	Wind tunnel test	<2% error between calculated and experimental thrust
	Aerodynamic profile optimization	[32]	HBC-COMEA algorithm	5.07% torque increase, 24% vibration reduction
	Rotor upscaling and load control	[26]	Sizing calculations and ALC	Fatigue load reduction and lower capital costs
	Blade shape optimization	[28]	Genetic Algorithm (GA)	10% increase in power output
	Endplate design comparison	[29]	CFD + experiments	Circular endplate performed best
	CAWT performance improvement	[33]	Optimized Deflector Cone (ODFC)	Power coefficient doubled
	Savonius turbine optimization	[30]	Taguchi + ANOVA	Inner blade angle most critical
Control Strategies and Smart Systems	Yaw control in wind farms	[35]	Dual yaw strategy	Output increased by 11.3%
	AI in turbine design	[36]	Review of digital twin, loads, standards	AI enables scalable, sustainable design
	Coordinated control (pitch/speed)	[37]	Multi-objective adaptive fuzzy control	Robust, dynamic system performance
	Blade damping via pitch control	[38]	Dynamic model	Effective power, speed and load control
	FOWT real-time control	[41]	RBFNN + Super-Twisting Algorithm	Improved stability in offshore conditions
	CCD of turbine systems	[40]	DFSM surrogate model	Tower stress limits critical to design
Fatigue, Vibration, and Structural Analysis	Structural fatigue mitigation	[44]	HSFD system	Reduced fatigue damage
	Vibration reduction strategies	[47]	Spectral analysis and simulation	Vibration reduction via blade-structure coupling
	Compression-bending capacity	[25]	Finite Element Model	Prediction error minimal (CoV = 0.0454)
	UHPC–steel hybrid tower design	[31]	Dynamic FE + cost analysis	Economically efficient hybrid design
	Impedance control and damping	[39]	Eigenvalue analysis	Stability drops as wind speed decreases
	Typhoon-induced loading	[46]	Dynamic simulation	Wind loads dominate extreme structural responses
Environmental Effects and Simulation	Atmospheric stability effect	[21]	3D Stability-COUTI + LES	High coherence in field vs. simulation data
	Wake flow in FOWTs	[43]	6-DOF dynamic model	Turbulence intensity increases with pitch amplitude
	Typhoon wind–wave forces	[42]	WRF-SWAN-FVCOM model	Pitch instability at 11.6° pitch angle
	Post-rated wind speed performance	[148]	CFD vs. BEM	CFD (RANS) more accurate than QBlade
Optimization and Efficiency Analysis	Offshore WT lifecycle analysis	[34]	Probabilistic failure prediction	Supports lifecycle decision-making
	WT generator system optimization	[24]	AMI-PSO algorithm	2.43% cost/kWh reduction, 5× cycle time cut
	HAWT hydrokinetic turbine blades	[22]	Taguchi + ANOVA	6% improvement in power coefficient
	Economic efficiency of tidal HAWT	[35]	Actuator disc model	Rotor shows high- and low-frequency fluctuations
	Hybrid renewable system design	[45]	PSO, WOA, ACO, GA	Alkhums, Libya: lowest COE achieved

**Figure A2:**
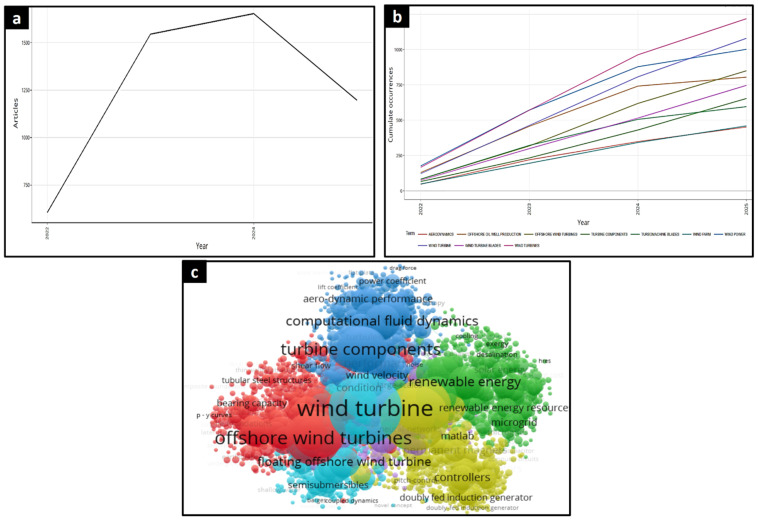
Thematic and temporal analysis. (**a**) Trend in annual scientific production from the initial unfiltered search. (**b**) Cumulative frequency of top keywords over time. (**c**) Co-occurrence network of keywords grouped into five thematic clusters.

## Data Availability

The original contributions presented in this study are included in the article. Further inquiries can be directed to the corresponding authors.

## Abbreviations

The following abbreviations are used in this manuscript:

ABC	Artificial Bee Colony
ACO	Ant Colony Optimization
AEP	Annual Energy Production
AI	Artificial Intelligence
ALC	Active Load Control
AMI-PSO	Adaptive Mutation Identity Particle Swarm Optimization
ANN	Artificial Neural Networks
ANOVA	Analysis of Variance
BEM	Blade Element Momentum
BiMADS	Bi-objective Mesh Adaptive Direct Search
CAWT	Cross-Axis Wind Turbine
CCD	Control Co-Design
CFD	Computational Fluid Dynamics
CFRPs	Carbon Fiber Reinforced Polymers
CoE	Cost of Energy
CoV	Coefficient of Variation
DFSM	Dynamic System Derivative Function Surrogate Model
DMTO	Discrete Material and Thickness Optimization
DOF	Degrees of Freedom
DTU	Technical University of Denmark
FEA	Finite Element Analysis
FEM	Finite Element Method
FOWT	Floating Offshore Wind Turbine
FSI	Fluid–Structure Interaction
FVCOM	Finite Volume Community Ocean Model
GA	Genetic Algorithm
GFRPs	Glass Fiber Reinforced Polymers
HAHT	Horizontal Axis Hydrokinetic Turbine
HAWT	Horizontal Axis Wind Turbine
HBC-COMEA	Hybrid Bidirectional Cooperative Constrained Multi-objective Evolutionary Algorithm
HSFD	Hinge-Spring-Friction Device
IEC	International Electrotechnical Commission
IoT	Internet of Things
LEP	Leading-Edge Protection
LES	Large Eddy Simulation
LSTM	Long Short-Term Memory
MDO	Multidisciplinary Design Optimization
NSGA-III	Non-dominated Sorting Genetic Algorithm III
O&M	Operations and Maintenance
ODFC	Omnidirectional Flow Concentrator
OWC	Oscillating Water Columns
OWT	Offshore Wind Turbine
PSO	Particle Swarm Optimization
QBlade	Open-source BEM-based wind turbine simulation tool
RANS	Reynolds-Averaged Navier–Stokes
RBFNN	Radial Basis Function Neural Network
ROS	Robot Operating System
RSM	Response Surface Methodology
SCADA	Supervisory Control and Data Acquisition
SDGs	Sustainable Development Goals
SHM	Structural Health Monitoring
SiC	Silicon Carbide
SMAs	Shape Memory Alloys
SST	Shear Stress Transport
STA	Super-Twisting Algorithm
SWAN	Simulating Waves Nearshore Model
TMDs	Tuned Mass Dampers
TSR	Tip Speed Ratio
UHPC	Ultra-High-Performance Concrete
VARTM	Vacuum-Assisted Resin Transfer Molding
VAWT	Vertical-Axis Wind Turbine
WOA	Whale Optimization Algorithm
WRF	Weather Research and Forecasting Model

## Appendix A. Bibliometric Analysis Methodology

This appendix details the methodology used to analyze research trends in wind turbine technology from 2022 to 2025.

### Appendix A.1. Data Collection and Filtering

The literature search was conducted on 30 June 2025, using three primary databases: OpenAlex, Scopus, and Dimensions AI. A combination of broad and specific search strings was used to capture a comprehensive set of relevant publications, as detailed in Table A1.

**Table A1:** Search strategy and results.

Database	Search Strings	Results	Export Format
OpenAlex	“Challenges in design of wind turbines”	1	CSV via Publish or Perish
Scopus	“Design” AND “Wind turbines”	5	CSV
Dimensions AI	“Wind turbines” AND “Structural analysis”	501	CSV
Total		6501	
Duplicate records (removed)		5445	
Final records		1056	

The initial search yielded 6501 results. After removing 5445 duplicates using datablist software (https://www.datablist.com/), the dataset was further refined based on the criteria in Table A2. This resulted in a final corpus of 1056 full research articles for analysis, which aligns with the data dashboard shown in Figure A1a.

**Table A2:** Data inclusion and exclusion criteria.

Inclusion Criteria	Exclusion Criteria
Published between 1 January 2022 and 30 June 2025	Articles published outside the specified date range
Full research articles only	Reviews, letters, notes, book chapters, conference papers, and errata
Must include search keywords in title or abstract	Articles without an abstract

### Appendix A.2. Analysis and Visualization

The bibliometric analysis was performed using the Bibliometrix R package and VOSviewer (v. 1.6.20) to map keyword trends and relationships. Figure A1 provides a multi-faceted overview of the final dataset. The main dashboard (a) summarizes the corpus of 1056 documents, highlighting key metrics such as an annual growth rate of over 25% and contributions from 11,600 authors. The word cloud (b) and treemap (c) both visualize keyword prominence, clearly identifying wind turbines, offshore wind, and wind power as the most dominant themes in the recent literature. Figure A2 presents a deeper analysis of thematic and temporal trends. The line graph (a) illustrates the strong growth in annual scientific production from the initial, unfiltered search results. The graph of cumulative frequencies (b) shows the steady rise in publications for the top keywords over the analysis period. Finally, the co-occurrence network (c) visualizes the relationships between keywords, grouping them into five distinct thematic clusters representing key research sub-fields such as aerodynamics, renewable energy integration, and structural components.
